# Surface Modification of Magnetic Iron Oxide Nanoparticles

**DOI:** 10.3390/nano8100810

**Published:** 2018-10-09

**Authors:** Nan Zhu, Haining Ji, Peng Yu, Jiaqi Niu, M. U. Farooq, M. Waseem Akram, I. O. Udego, Handong Li, Xiaobin Niu

**Affiliations:** 1 School of Materials and Energy, University of Electronic Science and Technology of China, Chengdu 610054, China; 18651927042@163.com (N.Z.); jiaqi_niu@163.com (J.N.); hdli@uestc.edu.cn (H.L.); xbniu@uestc.edu.cn (X.N.); 2 Institute of Fundamental and Frontier Science, University of Electronic Science and Technology, Chengdu 610054, China; Azarisyu@gmail.com (P.Y.); ufbajwa@yahoo.com (M.U.F.); waseem.physicist@gmail.com (M.W.A.); Israel.udego@yahoo.com (I.O.U.)

**Keywords:** magnetic nanoparticles, nanomedicine, iron oxide nanoparticles, surface modification

## Abstract

Functionalized iron oxide nanoparticles (IONPs) are of great interest due to wide range applications, especially in nanomedicine. However, they face challenges preventing their further applications such as rapid agglomeration, oxidation, etc. Appropriate surface modification of IONPs can conquer these barriers with improved physicochemical properties. This review summarizes recent advances in the surface modification of IONPs with small organic molecules, polymers and inorganic materials. The preparation methods, mechanisms and applications of surface-modified IONPs with different materials are discussed. Finally, the technical barriers of IONPs and their limitations in practical applications are pointed out, and the development trends and prospects are discussed.

## 1. Introduction

Recently, magnetic nanoparticles is an emerging field of study and has gained much attention among researchers due to their widespread applications in various fields including catalysis [1], data storage [2], environmental remediation [3], magnetic fluids [4], electronic communication [5], and biomedicine [6] etc. Among different types of magnetic nanoparticles (MNPs), iron oxide nanoparticles (IONPs) are the most popular and widely used in the field of biomedicine due to their ease of surface modification, synthesis, and low toxicity [7]. Current studies and literature have confirmed that magnetic IONPs are frequently used in the treatment of hyperthermia [8,9,10] or as drug carriers in cancer treatment [11,12,13], magnetic resonance imaging (MRI) agents [14,15,16], bioseparation [17,18,19], gene delivery [20,21,22], biosensors [23,24,25], protein purification [26,27,28], immunoassays [29,30,31] and cell labeling [32,33,34].

However, IONPs suffer from two major issues such as rapid agglomeration, oxidation into the physiological environment of the tumors due to large surface area, chemical reactivity and high surface energy, thus resulting in a loss of magnetism [35]. Therefore, appropriate surface modification of IONPs is required to make them biocompatible. The coating method is the most common surface modification approach to conjugate the organic or inorganic materials onto the surface of IONPs. This method not only prevents the oxidation and agglomeration of IONPs, but also provides the possibility for further functionalization [36]. Functionalization of magnetic IONPs can improve their physicochemical properties, making them ideal candidates for the field of catalysis and biomedicine.

Different characteristics such as size, shape, morphology and dispersability of the IONPs can affect their application in biomedicine [37,38]. Therefore, researchers are focusing on synthesizing MNPs by adopting different routes to control their size, shape and morphology with adjustable and desirable properties. So far, a number of synthesis routes such as co-precipitation [39], hydrothermal [40], thermal decomposition [41], microemulsion [42], electrochemical deposition [43], laser pyrolysis [44], solvothermal methods [45], sonochemical methods [46], chemical vapor deposition [47], the microwave assisted method [48], and aerosol pyrolysis [49] have been reported to prepare the magnetic IONPs. The advantages and disadvantages of some methods are listed in Table 1.

In this review, first we briefly describe the factors influencing why surface modification of MNPs is essentially required, and then introduce the structures of magnetic iron oxide nanocomposites. The materials used in surface modification are categorized as organic materials and inorganic materials. Organic material molecules are composed of small molecules and polymers while inorganic materials include silica, carbon, metals and metal oxides/sulfides. In next section, we summarize the IONPs’ surface coating mechanisms as well as the progress made in recent years, and highlight their applications in various fields.

## 2. Surface Modification of Magnetic Iron Oxide Nanoparticles (IONPs) and Applications

**Figure 1:**
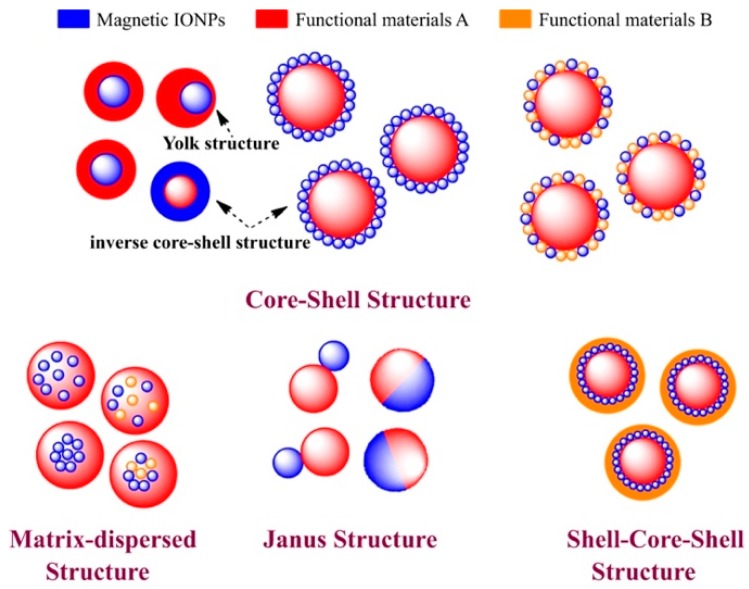
Typical morphologies of magnetic composite nanomaterials. Reproduced with permission from [50]. Copyright Institute of Physics, 2015.

There are four main purposes of surface modification of NPs: (1) to improve or change the dispersion of MNPs; (2) to improve the surface activity of MNPs; (3) to enhance the physicochemical and mechanical properties; and (4) to improve the bicompatibility of MNPs. There are mainly four magnetic iron oxide nanocomposites (Figure 1) [50]. 

### 2.1. Surface Coating with Inorganic Materials

#### 2.1.1. Silica

Silica is the most common and widely used agent for surface modification of IONPs [51,52,53,54,55]. Silica coating has following advantages: low agglomeration, enhancing the stability and reducing the cytotoxic effects of MNPs. Therefore, it has demonstrated good biocompatibility, hydrophilicity and stability [56]. Recently, researchers have described the procedure to control the size and thickness of the silica coated NPs [57]. Generally, there are four main approaches to prepare IONP@SiO_2_ (Table 2) [50].

The Stöber method is the most common approach to synthesize IONP@SiO_2_, in which the IONPs are uniformly dispersed in ethanol solution, followed by the addition of tetraethoxysilane (TEOS), then finally the aqueous ammonia solution is admixed to the mixed solution [50,58]. As a basic catalyst, ammonia can not only control the particle size, but also inhibit hydrolysis to form particles with regular morphology. Zhao Li et al. found that the size of silica particles increases with the concentration of ammonia, water, and TEOS in the reaction solution. At the same time, she found that an increase in the reaction temperature accelerated the ripening of the silica particles, causing the particle size to increase slightly [59]. This method can be applied to coat a SiO_2_ layer directly onto the surface of Fe_3_O_4_. Malvindi et al. studied the toxicity of silica-coated IONPs in a vitro model. They used the A549 and HeLa lines and incubated cells with surface-modified Fe_3_O_4_@SiO_2_ as well as bare NPs. They reported that the naked NPs show higher toxicity due to their stronger in situ degradation [60]. Uribe Madrid et al. demonstrated the synthesis of Fe_3_O_4_@mSiO_2_ core-shell structures with high specific surface area and different mesoporous silica (mSiO_2_) shell thickness. This composite nanoparticle synthesized via the modified Stöber method shows excellent drug release performance and it is ideal for targeted drug delivery in vivo [61].

**Figure 2:**
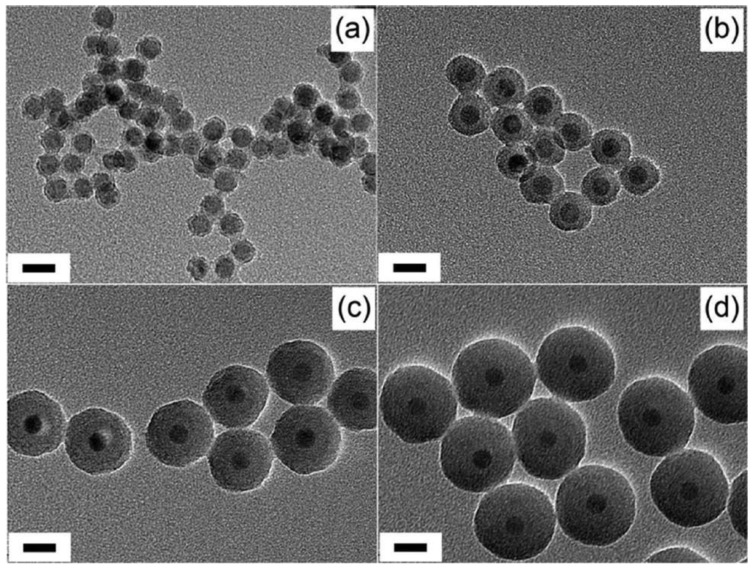
Transmission electron microscope (TEM) image of 12.2-nm Fe_3_O_4_@SiO_2_ nanoparticles (NPs) with shell thicknesses of (**a**) 2.0 nm, (**b**) 6.3 nm, (**c**) 14.1 nm, and (**d**) 19.8 nm. Scale bar = 20 nm. Reproduced with permission from [65]. Copyright American Chemical Society, 2012.

**Figure 3:**
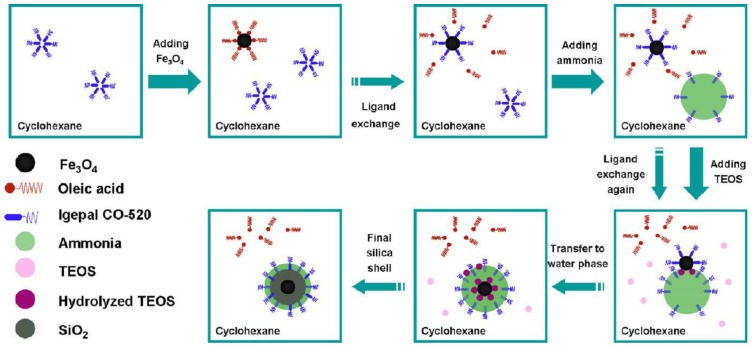
Illustration of the coating mechanism of SiO_2_ on the Surface of Fe_3_O_4_ NPs. Reproduced with permission from [62]. Copyright American Chemical Society, 2012.

The second method is the microemulsion method, which can be divided into two different types, namely water-in-oil (W/O, micelles) and oil-in-water (O/W, reversed micelles). Sillca-coated IONPs with high crystallinity can be synthesized by the microemulsion process, which comprises water, oil and surfactant [62]. Du et al. synthesized a silica-encapsulated Fe_3_O_4_ core-shell structure by the microemulsion approach and further functionalized with an antiseptic agent cetyl trimethylammonium bromide (CTAB). Their results have shown that the core size of Fe_3_O_4_ NPs depends on the water/surfactant molar ratio of the microemulsion system [63]. Yang et al. developed an oil–water two-phase layered coating strategy for the preparation of monodisperse dendritic mesoporous silica-encapsulated magnetic nanospheres with pore size of approximately 5.7 to 10.3 nm and shell thickness of 40 to 100 nm [64]. Some researchers have put forward their own views on the mechanism of silica coating in reverse microemulsion systems. Ding et al. summarized these viewpoints and found that it is generally accepted that there is a ligand exchange. They systematically studied the factors affecting the core size and shell thickness of Fe_3_O_4_@SiO_2_ NPs. They found that shell thickness increased with an increasing amount of ammonia and TEOS. Meanwhile, the small aqueous domain is suitable for ultrathin silica shell, while the large aqueous domain is essential for a thicker shell. Single-core Fe_3_O_4_@SiO_2_ NPs with different shell thicknesses are shown in Figure 2 and the surface coating mechanism is depicted in Figure 3 [65]. The microwave-assisted method can also be used to assist in the synthesis of Fe_3_O_4_@SiO_2_ NPs. Lu et al. prepared Fe_3_O_4_@SiO_2_ NPs with a very thin SiO_2_ shell (2.5 nm) by a novel microwave-assisted reverse microemulsion method [66]. The microemulsion method has the advantage of controlling the shape, size distributions and shell thickness. However, the poor yield and demand of large amounts of solvent are the major drawbacks of this method. The separation of NPs from surfactants is often time consuming and requires much effort [62].

**Scheme 1:**
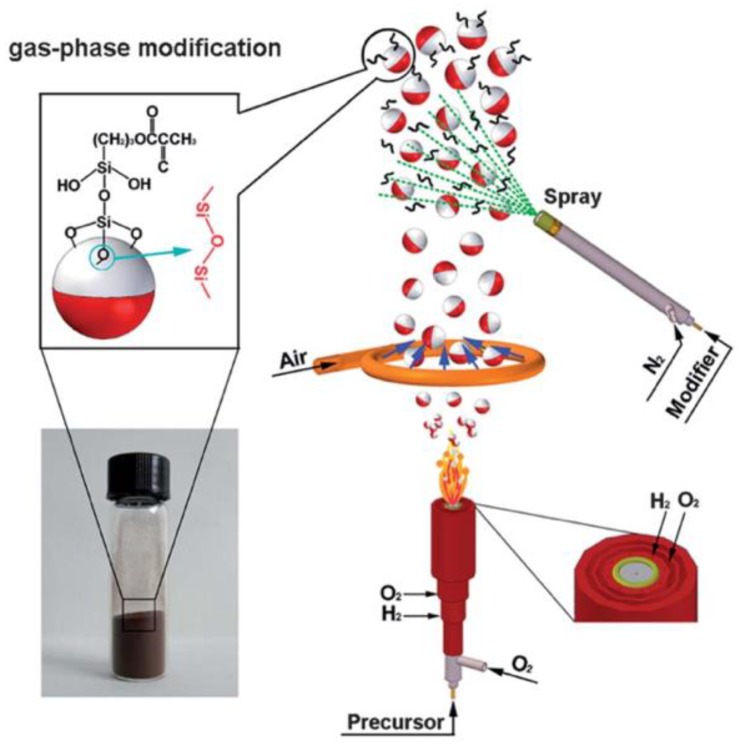
Schematic illustration of flame synthesis and in situ selective modification of double-faced g-Fe_2_O_3_||SiO_2_ NHs. Reproduced with permission from [67]. Copyright Royal Society of Chemistry, 2013.

The third method is aerosol pyrolysis, which is very innovative, highly productive and usually carried out in a flame environment [50,67]. Flame aerosol technology is widely used in large-scale production of carbon black and ceramic products such as fumed silica and titania [68], as well as zinc oxide and alumina powders [69,70]. Professor Pratsinis’ group has done a lot of research and made outstanding contributions in this field [69,71]. He developed the flame spray pyrolysis process for the aerosol synthesis of films and particles up to 5 kg/h in his labs. He has shown experimentally how to closely control aerosol particle size, crystallinity and morphology, from perfectly spherical to highly ramified fractal-like structures. Recently, his group published an article about the impact of humidity on silica nanoparticle agglomerate morphology and size distribution [72]. He studied scalable flame synthesis of SiO_2_ nanowires [68]. For example, Teleki et al. synthesized the IONPs by flame spray pyrolysis of acetylacetone iron in a xylene/acetonitrile solution, then in situ coated the resulting aerosol with SiO_2_ by oxidation of swirling hexamethlydisiloxane vapor. They eventually obtained hermetically-coated superparamagnetic Fe_2_O_3_ NPs with a relatively low SiO_2_ content [73]. Li et al. reported double-faced γ-Fe_2_O_3_@SiO_2_ nanohybrids with a “Janus” structure synthesized by a flame aerosol route. The aerosol route steps are illustrated in Scheme 1 [67].

The fourth method is based on sodium silicate solution, which is used as a silica precursor [74,75]. Setyawan et al. reported a strategy for synthesizing silica-coated magnetite NPs by the one-step electrochemical method. In the experimental system, sodium silicate serves as a dispersant and supporting electrolyte which helps to improve the conductivity of the solution. The anode and cathode of a chemical cell consists of iron and iron base, respectively [74,76]. Fajaroh et al. found that the concentration of sodium silicate solution has a positive and negative correlation with the crystallinity and surface area of the NPs, respectively [77].

Finally, a large number of reactive silanol groups present on the silica layer can be used for further surface functionalization [78]. (3-aminopropyl)triethoxysilane (APTES), (3-Mercaptopropyl)triMethoxysilane (MPTS), Triethoxy vinyl silanes (VTES), aminosilane are the most commonly used binding ligands. Functionalized Fe_3_O_4_@SiO_2_ MNPs have great applications in biomedicine and environmental fields [79,80,81].

#### 2.1.2. Carbon

Carbon-based materials as an inorganic compound are also used in surface coating of IONPs, to enhance their stability, biocompatibility and disperstivity. The Fe_3_O_4_@C nanocomposites have various applications, such as use as catalysts, electrode supercapacitors, microwave absorbers, anode materials for lithium-ion batteries, and so on [82,83,84,85,86].

Many research groups have shown that Fe_3_O_4_@C nanocomposites are the superior materials for supercapacitors [87]. For instance, Liu et al. synthesized carbon-coated Fe_3_O_4_ nanorods via hydrothermal reactions followed by a carbon-thermal reduction process, and demonstrated that Fe_3_O_4_/C nanorods exhibit higher specific capacitance as well as better cycle performance to that of pure Fe_3_O_4_. Generally this phenomenon arises due to the presence of a carbon layer that makes the particles intact and increases the electronic conductivity of the electrodes [88]. Sinan et al. synthesized Fe_3_O_4_ NPs by a co-precipitation method first and then successfully prepared layered porous Fe_3_O_4_/C nanocomposites with high specific surface area by hydrothermal carbonization and the MgO template method. This showed that Fe_3_O_4_/C nanocomposites are very promising for applications as a negative material for asymmetric supercapacitors [89]. Research shows that the presence of activated carbon with a three-dimensional (3D) network structure can enhance the conductivity and cycling stability of Fe_3_O_4_ [90].

Fe_3_O_4_@N-doped carbon NPs have great importance in lithium-ion batteries as an efficient oxygen-reduction electrocatalyst [91,92]. Number of materials can be used as N and C sources. For example, Yang et al. fabricated yolk–shell Fe_3_O_4_@Void@C–N NPs by using melamine formaldehyde resin [93]. Similarly, Yang et al. prepared Fe_3_O_4_@void@N-doped carbon with a yolk–shell structure where C and N are provided by ionic liquids. The introduction of nitrogen helps to enhance the lithium ion storage capacity, and the void acts as a buffer during the charging and discharging process [94]. Fe_3_O_4_@N-doped carbon NPs can also be used as an oxygen-reduction electrocatalyst [95,96].

Carbon nanotubes are one-dimensional nanomaterials made by curling one or more layers of graphite sheets in a certain way. Carbon nanotubes are widely fabricated as nanocomposites because of their metal-semiconductor characteristics, high mechanical strength, excellent adsorption capacity and microwave absorption capacity [97,98,99,100]. Guo et al. synthesized Fe_3_O_4_/CNTs nanocomposites with particle size of 80 nm via the hydrothermal method by using Sn(OH)_6_^2−^ as an inorganic dispersant. After 50 cycles, the Fe_3_O_4_/CNTs nanocomposites can provide reversible discharge capacity of 700 mAh/g at the 50 mA/g current density [101]. Zhu et al. synthesized Fe_3_O_4_/CNTs nanocomposites with 3D network by anchoring porous Fe_3_O_4_ spheres onto carbon nanotubes. This nanocomposite exhibits a remarkable microwave absorption property [102]. Zhang et al. prepared Fe_3_O_4_/CNTs nanocomposites by thermal decomposition of polyols, and conjugated these with hexanediamine and used them as a dual-drug carrier for the co-delivery of epirubicin hydrochloride and paclitaxel. Their designed Fe_3_O_4_/CNTs nanocomposites have potential applications in cancer treatment as a combined chemotherapy approach [103].

**Scheme 2:**
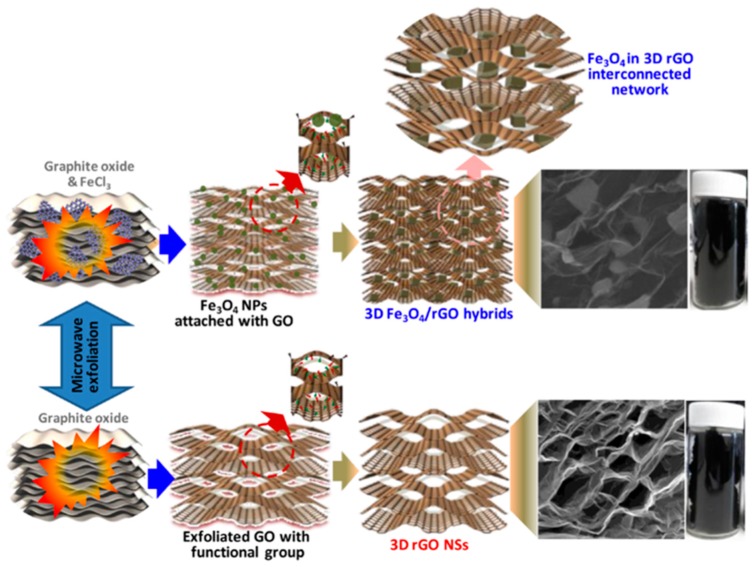
Schematic formation mechanism of three-dimensional Fe_3_O_4_/rGO hybrids. Reproduced with permission from [113]. Copyright American Chemical Society, 2017.

Fe_3_O_4_/graphene nanocomposites have drawn much attention in recent years [50,104]. Their superior properties have seen them widely applied in lithium batteries, ion removal, dye removal, catalysts, sensors, supercapacitor electrodes, microwave absorption, MRI, etc. [105,106,107,108,109,110,111,112]. Kumar et al. synthesized a 3D hybrid material composed of Fe_3_O_4_ NPs and reduced graphene oxide (rGO) nanosheets, in which Fe_3_O_4_ nanoparticles were introduced into the network of rGO nanosheets, and the formation mechanism is shown in Scheme 2. The specific capacitance of this hybrid material is determined by the surface morphology and interconnection of the two components [113]. A novel sandwich-structured graphene-Fe_3_O_4_ composites (graphene-Fe_3_O_4_@carbon) were prepared by Zhao et al., with higher reversible capacity, better cycling/rate performance than carbon-coated Fe_3_O_4_. The carbon layer serves as a buffer, while avoiding the aggregation of nanoparticles [114].

#### 2.1.3. Metal

Surface modification of IONPS with metallic elements can provide an inert layer, which typically exhibit a core-shell, core-satellite or dumbbell structure. Metallic coatings facilitate further functionalization of the IONPS to improve stability and compatibility [115]. 

**Scheme 3:**
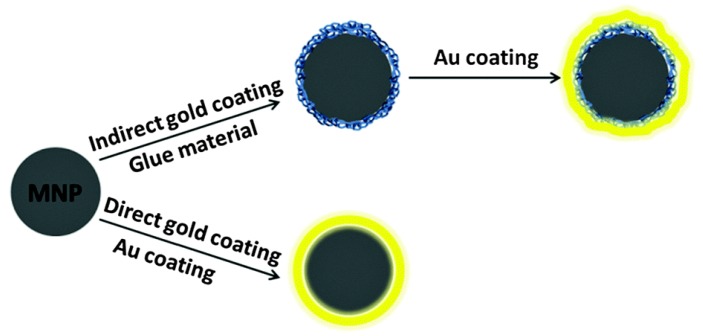
Schematic illustration of the two routes for gold coating. Reproduced with permission from [120]. Copyright Royal Society of Chemistry, 2016.

Gold is the most common noble metal element used for surface coating [116,117,118,119]. In general, direct and indirect method are the two routes to achieve the gold shell coating on the surface of the magnetic IONPs, as shown in Scheme 3 [120].

**Scheme 4:**
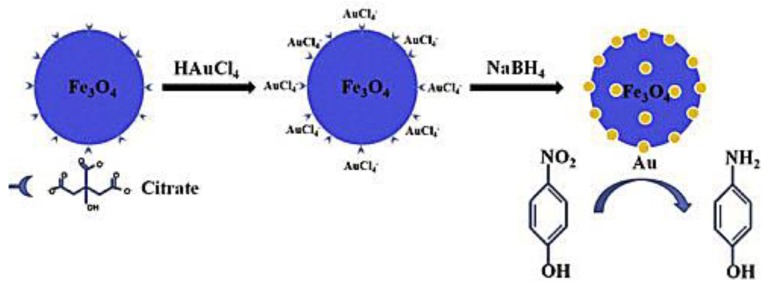
Schematic illustration of the bifunctional Fe_3_O_4_/Au nanocomposites. Reproduced with permission from [124]. Copyright Elsevier, 2014.

A direct method is to directly form a gold shell onto the surface of IONPs via reduction of Au_3_^+^ by using reducing agents. Direct gold coating can be carried out in aqueous or organic solution. In the aqueous phase, the sodium citrate and sodium borohydride are the frequently used reducing agents [116,121]. Ghorbani et al. synthesized the citrate-protected Fe_3_O_4_/Au NPs by the method of Lo. First, HAuCl_4_ was added to deionized water and heated to boiling. After that, the prepared Fe_3_O_4_ nanoparticle solution was added, followed by the insertion of sodium citrate under stirring. Finally, the mixture was boiled under stirring for 5 min and in doing so the solution color changed from brown to burgundy [122,123]. Yan et al. synthesized the carboxylate-functionalized Fe_3_O_4_ NPs by a one-step method and subsequently mixed it with HAuCl_4_ aquatic solution. Then added NaBH_4_ to the mixed solution to directly reduce the HAuCl_4_. Inspired by the experimental results, they put forward a mechanism to synthesize bifunctional Fe_3_O_4_@Au nanocomposites, as shown in Scheme 4. Carboxylic acid groups adsorbed AuCl_4_ under acidic condition, and then NaBH_4_ was added to form zero-valent Au which attached to Fe_3_O_4_ NPs through the chemistry of the carboxylate group, and gradually the gold shell was formed around Fe_3_O_4_ NPs [124]. Hydroxylamine hydrochloride is another reducing agent used [125].

In the organic synthesis route, the oleic acid and oleylamine are usually present as a capping agent in the solution. Freitas et al. used 1-hexadecanol to reduce Fe(acac)_3_ for amine-functionalized Fe_3_O_4_ NPs in the presence of oleic acid and oleylamine. Oleylamine also works as a reducing agent. These amine groups can attach Au^3+^. Then, they synthesized core-shell Fe_3_O_4_@Au MNPs in three Fe_3_O_4_:HAuCl_4_ molar ratios (1:1; 1:4; 1:7). Their results show that the gold shell formed in a ratio of 1:1 cannot completely encase the Fe_3_O_4_ core, while Fe_3_O_4_@Au obtained in a 1:4 ratio has the best performance [126]. Li and co-workers used the FeO(OH) and HAuCl_4_ as iron precursor and gold precursor, respectively. With the presence of oleic acid, octahedron-like Au/Fe_3_O_4_ NPs were synthesized by reducing FeO(OH) and HAuCl_4_ in 1-octadecene solvent. The size of the synthesized particles is controlled by the proportion of the starting materials [127].

**Scheme 5:**
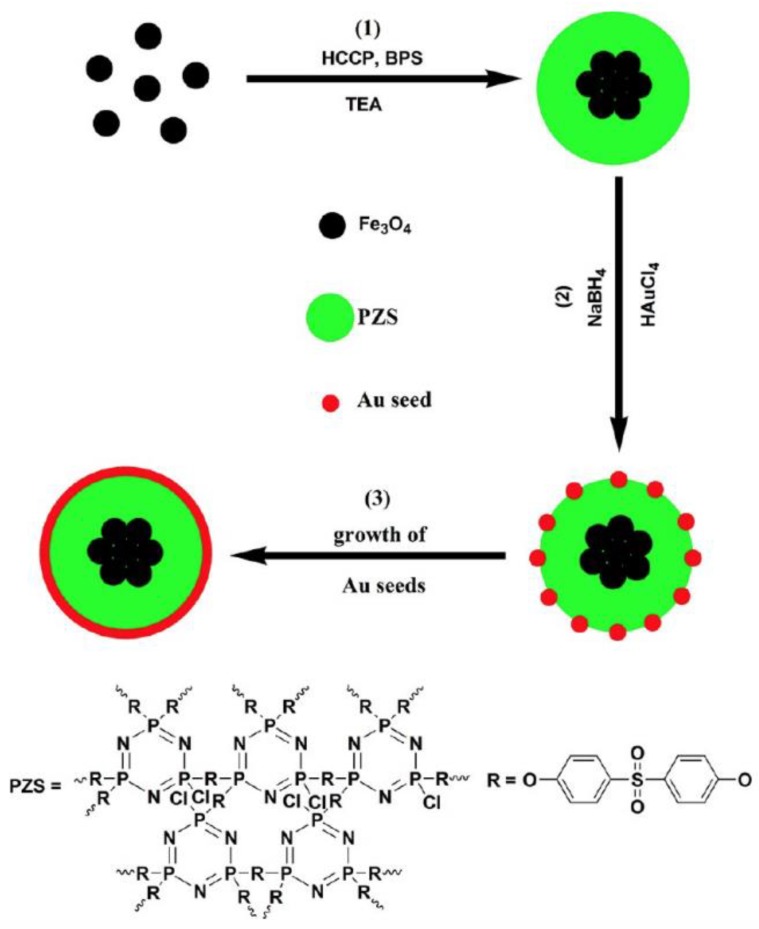
Preparation procedure of Fe_3_O_4_@PZS@Au shells. Reproduced with permission from [131]. Copyright American Chemical Society, 2013.

An indirect method is used to synthesize Fe_3_O_4_@Au NPs by forming a “glue” layer between the IONPs core and the gold shell. The layer design and preparation are crucial during the synthesis as they can affect the properties of the Fe_3_O_4_@Au NPs [128]. The “glue” layer should be capable of enhancing the Fe_3_O_4_ MNPs stability, and also have metal binding groups to attach gold seeds to promote the formation of gold shell. Materials used as the “glue” layer are often polymers, silica and carbon [129,130,131,132]. Wang et al. used the mercapto-silica shell as the “glue” layer combining the Fe_3_O_4_ core with the Au shell and obtained the durian-like multifunctional Fe_3_O_4_@Au nanocomposites [129]. Li et al. synthesized amino-functionalized Fe_3_O_4_@SiO_2_ NPs firstly and then reduced HAuCl_4_ by a seed growth method to obtain Fe_3_O_4_@Au NPs [130]. Polyphosphazene (PZS) as the “glue” layer has been studied. The steps involved in the preparation are shown in the Scheme 5. These synthesized Fe_3_O_4_@PZS@Au NPs has multiple functions for MRI and photothermal therapy, as well as the potential to be used as a biosensor and catalyst [131]. Ramos-Tejada et al. developed a new approach to synthesize Fe_3_O_4_/Au NPs. The first step involved mixing FeSO_4_ and distilled water, by adding KNO_3_ and NaOH in an oxygen-free environment at 90 °C to synthesize Fe_3_O_4_ core. The second step involved mixing MNP suspension with polyethylenimine (PEI) solution, by applying sonication to achieve a first PEI layer; subsequently a second poly(styrenesulfonate) (PSS) layer and a third PEI layer were added using a layer-by-layer techniques. The third step involved using NaBH_4_ to reduce chloroauric acid to obtain negatively charged gold NPs. In a fourth step, the previously obtained polymer-coated NPs were dispersed in water until the particle concentration was 0.25 mg/mL, by adding the resulting suspension dropwise to the gold seed solution, after sonication and subsequent further treatment, Fe_3_O_4_/Au NPs were finally obtained [133].

Silver is another noble metal used for surface coating. Silver coated magnetic (Fe_3_O_4_@Ag) NPs have appealing applications in the field of biomedicine [134,135]. Chen et al. prepared the Ag@Fe_3_O_4_ nanowire by the solvothermal method and the showed that these NPs possessed peroxidase-like catalyst activity, which makes them suitable candidates for biomedicine [136]. Gao et al.’s experiments on mice demonstrated that Fe_3_O_4_@Ag hybrid NPs are effective computed tomography (CT) contrast agents, and thus can be used for in vivo CT imaging [137]. Du et al. reported a portable SERS (surface enhanced Raman scattering) sensor based on Fe_3_O_4_@Ag core-shell NPs to distinguish arsenic species [138]. 

On the other hand, silver-coated magnetic nanocomposites are considered as promising multifunctional materials as well because they have unique antibacterial characteristics [139,140,141,142]. However, hybrid Fe_3_O_4_@C@Ag are the dominant class of nanocomposites owing to their widespread applications in different research areas [143,144,145]. Xia et al. synthesized Fe_3_O_4_@C@Ag nanocomposites and concluded that by introducing a carbon layer, their synthesized nanocomposites have better antibacterial activity compared to Fe_3_O_4_@Ag. Thus the hybrid Fe_3_O_4_@C@Ag can be used as catalysts, antibacterial agents, adsorbents and bi-functional magneto-optical probes [146]. Chen et al. reported a multifunctional system based on Fe_3_O_4_@C@Ag hybrid NPs that can be used as a bi-functional probe for MRI and two-photon fluorescence (TPF) imaging techniques as well as near-infrared light responsive drug delivery [147].

#### 2.1.4. Metal Oxides/Sulfides

**Scheme 6:**
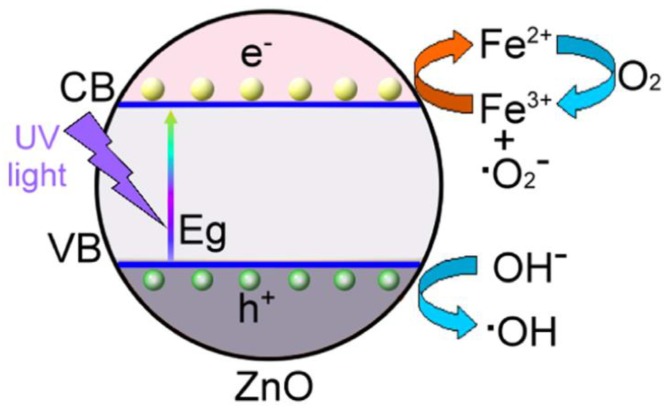
A proposed photocatalytic reaction mechanism of the Fe_3_O_4_@ZnO. Reproduced with permission from [150]. Copyright Elsevier, 2014.

Distinctive physicochemical properties of metal oxides and metal sulfides are of great interest in the functionalization of IONPs [50,148]. Generally, by considering the structure, the metal oxides are divided into six main categories such as M_2_O (Cu_2_O, Ag_2_O etc.), MO (ZnO, MgO, CoO, ZnS, CdS etc.), M_2_O_3_ (Al_2_O_3_, Y_2_O_3_, Bi_2_S_3_ etc.), MO_2_ (TiO_2_, SnO_2_ etc.), M_2_O_5_ (V_2_O_5_ etc.) and MO_3_ (WO_3_, MoO_3_ etc.). Saffari prepared superparamagnetic Fe_3_O_4_-ZnO nanocomposites with 10% ZnO content by adopting the sonochemical method and reported that Fe_3_O_4_/ZnO nanocomposite has excellent photocatalytic properties. Through the degradation analysis of eight kinds of organic dyes, it was found that Fe_3_O_4_/ZnO nanocomposite has suitable photocatalytic properties. Moreover, the Fe_3_O_4_@ZnO core/shell NPs have enhanced photocatalytic performance compared to bare ZnO NPs [149]. Wang’s group prepared the reusable Fe_3_O_4_@ZnO core/shell NPs and studied their photo-catalytic characteristics. According to the photocatalytic reaction mechanism of the Fe_3_O_4_@ZnO (Scheme 6), they attributed this phenomenon to the higher surface oxygen vacancy concentration and the inhibition of photo-induced electron-hole pairs recombination by Fe^3+^ ions [150]. Recently, Shekofteh-Gohari et al. fabricated a new type of magnetically separable Fe_3_O_4_/ZnO/CoWO_4_ nanocomposites with a different ratio of CoWO_4_. When the ratio of CoWO_4_ is 30%, the nanocomposites showed excellent photocatalytic activity. In terms of the degradation rate constant of rhodamine B, the optimum nanocomposites were 24 and 5 times higher than in the absence of laser source or the untreated samples of Fe_3_O_4_/ZnO and Fe_3_O_4_/CoWO_4_ samples, respectively [151]. In addition, Fe_3_O_4_/Al_2_O_3_ NPs can be used as adsorbents to remove ions in water. Doping with sulfate can be used to remove F^-^ in drinking water. A fluoride adsorption process was rapid in the beginning while slower as the passage of time; nearly 90% adsorption was achieved within 20 min [152]. Similarly, in another study, the Fe_3_O_4_@TiO_2_ core-shell magnetic composites were used as sorbents to efficiently sorb uranium (VI) [153]. Moreover, Liu et al. found that the Fe_3_O_4_@TiO_2_ with a yolk-shell structure has enhanced microwave absorption performance than that with a core-shell structure [154].

Generally, the combination of hard and soft magnetics will lead to many new applications [155]. Hard-soft magnetic composites are widely used in permanent magnets, data storage systems, image contrast agents and microwave devices [156]. In these bi-magnetic nanostructures, the size of the soft phase often determines the magnetization switch behavior. A typical example is the combination of hard magnetic CoFe_2_O_4_ and soft magnetic Fe_3_O_4_ [157,158]. Zeng et al. reported that composites (CoFe_2_O_4_/Fe_3_O_4_) have larger remanence (Mr/Ms), coercivity (Hc) and maximum energy product (BH)max than pure ferrite (CoFe_2_O_4_) due to exchange coupling [156,159].

### 2.2. Surface Coating with Organic Materials

#### 2.2.1. Polymers

In recent years, polymer-coated IONPs have drawn much more attention owing to their widespread applications in various research areas including nanomedicine. In situ and post-annealing coating are two common approaches to synthesize polymer coated IONPs [78]. The former is coating the polymer onto the surface of IONPs during the synthesis process [160]. The latter is further polymer functionalized on the basis of previously prepared IONPs. Todate, dextran, chitosan, alginate, polyethylene glycol (PEG), polyvinyl alcohol (PVA), polydopamine (PDA), polysaccharide, polyethylenimine, polyvinylpyrrolidone (PVP), poly acid polyetherimide, and polyamidoamine (PAMAM) are the most commonly used polymers for the surface modification of IONPs (Table 3) [161,162,163,164,165,166,167,168,169,170,171].

Dextran is a polysaccharide with excellent biocompatibility as well as good water solubility and its coating onto the IONPs has an impact on their physicochemical properties. Shaterabadi et al. found that dextran coating reduces the saturation magnetization of the IONPs which is mainly due to the presence of dextran non-magnetic shell. Moreover, the dextran coating also reduces the cytotoxicity of the IONPs, therefore making the nanocarrier excellent while enhancing their biocompatibility [160]. In addition, Hauser et al. found that synthetic methods, the amount of dextran can greatly affect the properties of the IONPs such as size, stability, crystallinity and magnetism [172]. Owing to its biosafety, bioactivity, biocompatibility, low cytotoxicity, dextran-coated IONPs are considered promising candidates for biomedical applications [173]. Unterweger et al. developed a novel drug delivery system by coating IONPs with dextran and cisplatin hyaluronic acid. After testing in the Jurkat cell line and the PC-3 cell line, the drug-free IONPs showed good biocompatibility and no cytotoxic effects, whereas the IONPs incorporated with cisplatin were able to induce apoptosis [174]. Osborne et al. synthesized dextran-coated IONPs which can be used as clinical MRI contrast agents in two-step and one-step procedures with the aid of microwaves. This method is simple, versatile, cost effective and repeatable. Therefore, the complexity of manufacturing processes is greatly resolved which makes the commercial production of surface modified IONPs possible [161].

**Scheme 7:**
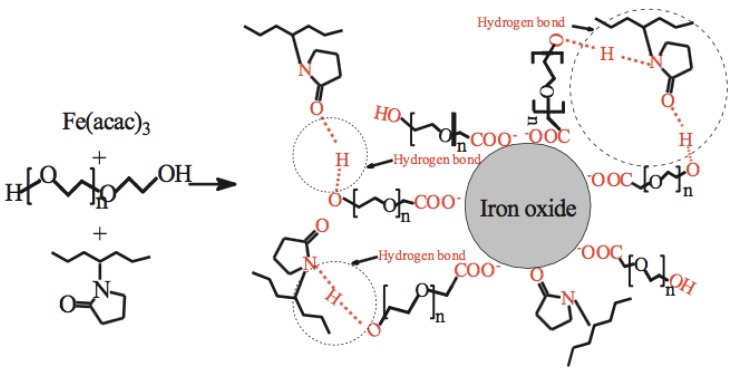
Schematic illustration of the synthesis of PEG/PVP-coated superparamagnetic iron oxide nanoparticles (SPIONs). Reproduced with permission from [180]. Copyright Elsevier, 2013.

PEG is another frequently-used water-soluble polymer. In the past, several methods and approaches have been reported to synthesize PEG-coated IONPs for the purpose of biomedical applications [164,175,176,177]. Liu et al. developed a simple strategy in which rich carboxyl groups were introduced through the multiple coordination between poly(acrylic acid) (PAA) and IONPs, then α, ω-diamino PEG was attached to IONPs by amidation of carboxyl groups. In vitro experiments showed that these surface-decorated IONPs significantly attenuated macrophage phagocytosis, as described by Lee’s group [178,179]. Moreover, it is also reported PEG-coated Fe_3_O_4_ NPs can prevent the reduction of cytochrome C [175]. Anbarasu et al. obtained Fe_3_O_4_ with a cubic inverse spinel structure by the coprecipitation method and found that both the average crystallite size and physical size of the NPs were decreased by increasing the amount of PEG [164]. Another synthesizing approach has been reported to prepare superparamagnetic IONPs with tunable properties using PEG containing PVP or PEI. An illustration of the synthesis of PEG/PVP-coated superparamagnetic iron oxide nanoparticles (SPIONs) is illustrated in Scheme 7. The prepared superparamagnetic IONPs had a hydrodynamic size less than 40 nm, with neutral or positive zeta potentials, showed higher dispersion stability than those IONPs coated with PEG alone [180].

Chitosan is an alkaline hydrophilic polymer whose low toxicity, good biocompatibility and biodegradability is confirmed in reported work [162,181,182]. Chitosan-coated IONPs are usually further functionalized with other polymers such as PEG and PAA [183,184]. For instance, Yan et al. prepared chitosan-PAA coated magnetic composite microspheres and found that the addition of PAA significantly increased the adsorption capacity of Cu (II) [183]. Qu et al. loaded 10-hydroxycamptothecin (HCPT) onto prepared PEG-chitosan-Fe_3_O_4_ nanocomposites. The synthesis steps of PEG-chitosan-Fe_3_O_4_ nanocomposites are as follows. The chitosan-Fe_3_O_4_ was first dispersed in phosphate buffered saline. 1-ethyl-3-(3-dimethylaminopropyl) carbodiimide hydrochloride was then added to activate the carboxylic acid moiety of the subsequently added carboxymethylated PEG (CM-PEG). Next, the reaction mixture was stirred and cultured at room temperature for 48 h. Thereafter, unreacted CM-PEG was removed by centrifugation. Finally, PEG-chitosan-Fe_3_O_4_ was washed three times with deionized water, collected by magnetic separation and lyophilized. Compared to the original HCPT powder, the HCPT-loaded nanocomposites showed higher antitumor activity against HepG_2_ cells. Besides, these nanocomposites can also be used for targeted hyperthermia [184]. However, the use of pure chitosan is limited due to its low solubility in acidic averments, low mechanical and thermal stability. Therefore, the researchers are trying to use the chitosan derivatives in order to overcome the aforementioned drawbacks of pure chitosan. N, O-carboxymethyl chitosan (CC) as a kind of chitosan carboxylation product has a good application prospect in membrane-forming. The membrane modified with CC-Fe_3_O_4_ NPs has improved hydrophilicity and anti-fouling properties [185].

Other polymers like polydopamine, polysaccharides, polylactic acid, polyacrylic acid, alginate, polyvinylidene fluoride, PEI, PVP, and PAMAM are also commonly used in surface conjugation of IONPs [186,187,188]. Recently, a new method based on cathodic electrochemical deposition (CED) and in situ coating was developed to prepare polysaccharide-coated Fe_3_O_4_ NPs [167]. PEI-coated IONPs can be further carboxylated or acetylated to enhance their biocompatibility [189]. PDA-coated Fe_3_O_4_ NPs can be used to detect small molecule pollutants [166]. Bian et al. formed Fe_3_O_4_@PDA-Pt composite by depositing Pt dendrimer-like NPs in situ on Fe_3_O_4_@PDA core-shell nanocomposites. This composite exhibited high catalytic performance for methylene blue, 4-nitrophenol and its derivative. It is worth mentioning that as a catalyst, it has good reusability and high stability [190]. Dimethyl sulfoxide (DMSO) can be used as a stabilizer to synthesize IONPs [191]. Yan et al. developed a novel potential MRI contrast agent by in situ synthesis of SPIO with immobilized SI-ATRP initiator and polymer analogs of DMSO. DMSO-based polymer acts to enhance the interaction between the MNPs and the water protons.

#### 2.2.2. Small Molecules and Surfactants

**Scheme 8:**
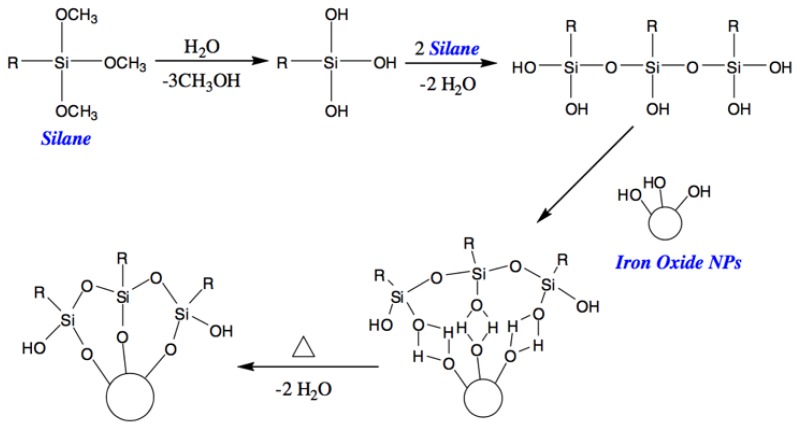
Physicochemical mechanism for modifying the silane agents on the surface of iron oxide NPs. Reproduced with permission from [155]. Copyright Wei Wu, 2008.

Functionalized NPs can be divided into three main types: lipophilic, hydrophilic and amphiphilic. This form of division is based on different surface characteristics of such surface-coated NPs [155]. Inorganic compounds such as silane as a coupling agents can be used to bind the different functional groups (e.g., –OH, –COOH, –NH_2_, –SH) onto the surface of bare MNPs and further their conjugation with different biomolecules, metal ions and polymers in order to make these MNPs suitable for various applications in diverse research areas [50]. The mechanism of IONPs modified by silane agents is shown in Scheme 8 [155]. Briefly, 3-aminopropyltriethyloxysilane (APTES), mercaptopropyltriethoxysilane (MPTES), and triethoxyvinylsilane (VTES) are the common silane coupling agents used in the surface modification of IONPs. Wang et al. adopted the sonochemical method to prepare the APTES-coated Fe_3_O_4_ NPs with diameter 8.4 ± 2.1 nm and concluded that the synthesized NPs exhibited superparamagnetism and good dispersibility. Magneto-rheological fluids prepared on the basis of these IONPs have typical magneto-rheological properties [192]. Li et al. developed acetylated APTES-coated Fe_3_O_4_ NPs based on the hydrothermal method. Based on their analysis results, they concluded that acetylation can improve the biocompatibility of NPs. The novel nanoparticle can be used for in vitro and in vivo MRI [193]. In addition, APTES functionalized magnetic iron oxide is also applied in the extraction of metal ions. In a study conducted by Mahmoud, it is reported that APTES functionalized Fe_3_O_4_ are capable of adsorbing Pb^2+^, Cu^2+^, Cd^2+^ and Hg^2+^ from aqueous solutions [194].

Lipophilic substances such as oleic acid are typically referred to as "fat-loving" or "fat-liking" and are of great interest for researchers to prepare lipophilic IONPs with very good dissolvability in polar liquids such as oil [195,196]. Additionally, oleic acid can form a dense protective monolayer that binds firmly to the NPs surface to stabilize the NPs [197]. Recently, the high degree of monodispersity, excellent biocompatibility, low toxicity, high colloidal stability and hydrophobicity of different types of IONPs having surface conjugation with oleic acid have been reported by many research groups. For example, Marinca et al. introduced a new synthetic route to prepare oleic acid-coated magnetite NPs. In the first step, iron and hematite were mixed and then heated. In the second step, the resulting magnetite powder and oleic acid are wet-mechanically milled. In their study, they did a comparison between the dry milling and wet milling NPs. Based on their experiment they concluded that the magnetite particles obtained by wet milling have a higher magnetization [198]. Velusamy et al. found that the conjugation of oleic acid onto the surface of IONPs can significantly reduce the growth and metabolism of biofilms and thus it can be used to inhibit the biofilm formation onto the surface of biomaterials [199].

However, in a biomedical scenario, the use of lipophilic substances coated IONPs is a not a good choice and thus the practical use of these NPs is greatly limited. To enhance the practical applications of IONPs, the research is focusing on synthesizing hydrophilic or water-soluble IONPs. Different organic molecules such as amino acids [200], citric acid [201], vitamins [202,203], cyclodextrin [204], dopamine [205,206], lauric acid [207], dimercaptosuccinic acid (DMSA) [208,209] are often used to modify the surface of IONPs by adopting different synthesis approaches so that the water solubility of IONPs can be greatly enhanced. One approach is to add these small organic molecules directly during the synthesis procedure. Jin et al. modified Fe_3_O_4_ NPs with arginine, lysine and poly-l-lysine, respectively. They found that these samples have high bacterial capture efficiency in the pH range 4–10 [200]. Recently, Karimzadeh et al. developed a novel method for the synthesis of amino acid modified Fe_3_O_4_ NPs based on CED [210]. Sahoo et al. have described that citric acid can adsorb on the surface of magnetite NPs through the coordination of one or two carboxylate functional groups [211]. Durdureanu-Angheluta et al. adopted the liquid laser ablation technique to synthesize citric acid-coated IONPs having spherical nature with an average size of about 60 nm. Their synthesized NPs have a core–shell structure and contain an outer layer of hydrophilic material of citric acid, which helps the NPs to stabilize in aqueous dispersions [212]. On the other hand, a cheap hydrophilic substance with lipophilic cavities, known as β-cyclodextrin is also used in the surface decoration of IONPs. In what follows, Li et al. prepared β-cyclodextrin modified Fe_3_O_4_ NPs by N_2_ plasma-induced grafting. This composite is suitable for the removal of organic and inorganic contaminants [204]. A few research groups also used the ascorbic acid (vitamin C) owing to their water solubility and anti-oxidant property, for surface modification of IONPs and concluded that these surface decorated NPs can be used as MRI contrast agents [203].

The hydrophobic nature of the IONPs can be changed to hydrophilic by adopting three different approaches, for example, amphiphilic polymer coatings [197], ligand exchange [213] and surface fatty acid oxidation [214]. Among them, ligand exchange involves an excess of ligand molecules, and the hydrophobic groups on the surface of the NPs are displaced by the ligand exchange reaction. Patil et al. further functionalized the oleic acid-coated Fe_3_O_4_ NPs with betaine-HCl (BTH) to create a new hydrophilic shell that ultimately resulted in the transfer from non-aqueous phase to aqueous phase. IONPs can also be transferred to aqueous solutions by oxidizing the oleic acid layer on the surface of NPs [215]. Cai et al. proposed a universal and efficient method for the large-scale transfer of hydrophobic Fe_3_O_4_ NPs to the aqueous phase [216]. This method is based on the oxidative decomposition of oleic acid in a reverse micelle system assisted by poly(vinylpyrrolidone) (PVP) [217]. The phase transfer process is as follows. Firstly, the prepared hydrophobic Fe_3_O_4_ NPs were dispersed in cyclohexane, and then tert-butanol, K_2_CO_3_, aqueous PVP solution and oxidizing agent were added and stirred at room temperature for 2 h. Finally, the NPs obtained were washed with ethanol and water for three times. The resulting NPs have superior features, like ideal colloidal stability, excellent biocompatibility, low cytotoxicity. Moreover, this phase transfer strategy also applies to other oleic acid-coated NPs [216].

## 3. Conclusions

This review summarizes the surface modification of IONPs by different organic molecules including surfactants as well as polymers and inorganic materials that include silicon groups, carbon, metal [218] and metal oxides/sulfides. Surface coating can improve the stability, biocompatibility, and even the solubility of IONPs, which greatly expands the scope of application of IONPs. Different synthesis methods, reaction mechanisms, performance, improvement and potential applications were also discussed. Important research findings in recent years are cited in this review, in the hope that this provides the readers a necessary background with logically coherent arguments about the surface modification of IONPs.

At present, although some progress has been made in the research on the modification of IONPs, several limitations still exist. Firstly, it is still a challenge to absolutely control the shape and size distribution of magnetic IONPs. Secondly, the issue of how to maintain the long-term stability of functionalized IONPs also needs to be addressed [50] Finally, most of the applications, especially those related to the clinical aspects, still remain in the theoretical stage and there is a long way to go before they can be applied in practice. Thus, much effort needs to be devoted to optimizing synthetic routes to obtain better IONPs. At the same time, it is essential to develop accessible, efficient, stable and environmentally friendly surface modification materials. Future research will focus on the multifunctional MNPs needed in clinical practice [57] In the future, with the improvement of surface modification technology and the development of surface modification materials, more and more multifunctional NPs will be developed and put into practical application.

## Figures and Tables

**Table 1:** Principal preparation methods of iron oxide nanoparticles (IONPs).

Method	Advantages	Disadvantages
Co-precipitation method	Simple and efficient	Size distribution, poor crystallinity and aggregation
Hydrothermal reactions	Easy to control particle size and shape	Long reaction time, high reaction temperature, high pressure
Thermal decomposition	Good control of size and shapes, high yield	High reaction temperature
Microemulsion method	Control of particle size, highly homogeneous	Poor yield, large amounts of solvent required and time consuming
Sol-gel reactions	Precise control of size and structure	Relatively expensive, long reaction time
Aerosol/vapor phase method	High yield	Extremely high temperatures
Electrochemical method	Easy control of size	Reproducibility

**Table 2:** Summary of synthesis methods for silica-coated IONPs.

Synthesis Methods	Advantages	Disadvantages
Stöber method	Controllable silica shell and uniform size, high crystallinity	Lack of understanding of its kinetics and mechanism
Microemulsion	Control of the particle size, high homogeneous	Poor yield, large amounts of solvent required and time consuming
Aerosol pyrolysis	Hermetically-coated	Complex experimental conditions
Methods based on sodium silicate solution	Control of crystallinity and surface area	Depends on preparation method

**Table 3:** Polymers used for coating IONPs and their applications.

Polymer	Source/Production/Preparation	Applications
Polyethylene glycol (PEG)	Produced by the interaction of ethylene oxide with water, ethylene glycol, or ethylene glycol oligomers	Magnetic resonance imaging (MRI) contrast agents for in vivo cancer imaging, biosensors
Polyvinylvinyl pyrrolidone (PVP)	Made from the monomer N-vinylpyrrolidone	Targeted killing of breast cancer cells, MRI contrast agents
Polyethylenimine (PEI)	Branched PEI: by the ring opening polymerization of aziridine Linear PEI: by post-modification of other polymers like poly(2-oxazolines) or N-substituted polyaziridines	Cancer cell separation, hyperthermia
Polyacrylic acids	Polymerization of acrylic acid	Anticancer drug delivery
Polyvinyl alcohol (PVA)	Polymerization of vinyl acetate, then saponification of polyvinyl acetate	In vivo imaging, drug delivery, biosensor
Polydopamine (PDA)	Formed from dopamine at slightly basic pH	Catalyst and adsorbent, biosensors
Dextran	Produced by lactic acid bacteria	In vivo cancer drug carriers, MRI contrast agents
Chitosan	Extracted from shellfish or fungi cell wall	Hyperthermia, tissue engineering
Starch	Produced by green plants	Contrasting and imaging
Alginate	Extracted from brown algae	Drug-targeted controlled release, adsorbent
Polyphenol	Found in some common plant foods like cocoa beans, tea and vegetables	Magnetic hyperthermia
Flavonoids	Found in some common plant foods like fruits, vegetables, beans and tea	Cell imaging, nano-carrier; nano-drug
Amino acids	In nature	Adsorbent, radio-labeling, biosensors and cancer detection
Lipids	In nature, animal food and nuts	Gene therapy, dual-modal imaging
